# Between protection and autonomy: a comparative analysis of legal approaches to abortion access for young people

**DOI:** 10.1080/26410397.2026.2712800

**Published:** 2026-08-07

**Authors:** Rebecca Reingold, Paulina Macías Ortega, Guillermina Pappier, Laura Dragnic Tohá, Sarah Wetter

**Affiliations:** a Associate Director, O'Neill Institute for National and Global Health Law, Georgetown Law, Washington, DC, USA.; b Former Research Assistant, O'Neill Institute for National and Global Health Law, Georgetown Law, Washington, DC, USA; c Former Fellow, O'Neill Institute for National and Global Health Law, Georgetown Law, Washington, DC, USA; d Former Research Assistant, O'Neill Institute for National and Global Health Law, Georgetown Law, Washington, DC, USA; e Senior Associate, O'Neill Institute for National and Global Health Law, Georgetown Law, Washington, DC, USA

**Keywords:** parental involvement, autonomy, evolving capacities, abortion, reproductive rights, progressive autonomy

## Abstract

This article analyses national legal frameworks that regulate young people's access to abortion, focusing on how different approaches to decision-making authority can either facilitate or obstruct access to care. It explores how countries that have recently liberalised their abortion laws, namely Colombia, Argentina, and Mexico, have acknowledged the need to lower the ages of consent below 18 and integrate the principles of the “evolving capacities of the child” or “progressive autonomy” into their legislation. Each country's approach, whether lowering the age of consent in specific circumstances (Mexico), establishing differentiated age brackets with varying degrees of autonomy (Argentina), or removing age-based restrictions altogether (Colombia), offers a different mechanism for increasing young people's autonomy in decision-making involving abortion. The article also considers how a country that has experienced a recent regression in abortion rights, namely the United States, has also rolled back young people's ability to autonomously consent to abortion care and increased parental involvement in such decisions. Through a comparative analysis of these four countries’ legal frameworks, the article concludes that sweeping changes to abortion law and policy – whether progressive or regressive in nature – can fundamentally alter young people's ability to consent to and access such care. It also issues a series of recommendations for the regulation of young people's abortion decision-making, including grounding relevant laws and policies in international human rights principles and linking them to laws and policies geared towards protecting young people's rights in general.

## Plain language summary

Parents or adults often help young people with medical decisions, including abortion care. However, international organisations and experts stress the need to respect young people's autonomy in these choices, especially regarding sexual and reproductive health care. Recently, countries like Colombia, Argentina, and Mexico have passed laws allowing young people to consent to abortion without parental or other adult involvement. This shows a recognition of their ability to make important decisions. In contrast, the United States has restricted young people's rights to make independent choices about abortion care. This article compares how these four countries handle young people's abortion decisions. It considers the broader legal and political context behind new laws and policies. It also looks at how reforms to general abortion laws and international human rights principles have influenced each country's approach, either supporting or undermining young people's reproductive rights and autonomy.

## Résumé

Cet article analyse les cadres juridiques nationaux qui régulent l’accès des jeunes à l’avortement, en s’intéressant particulièrement à la manière dont différentes approches du pouvoir de décision peuvent faciliter ou entraver cet accès. Il examine comment des pays qui ont récemment libéralisé leur loi sur l’avortement, à savoir la Colombie, l’Argentine et le Mexique, ont reconnu la nécessité d’abaisser l’âge du consentement en-dessous de 18 ans et d’intégrer les principes de « l’évolution des capacités de l’enfant » ou de « l’autonomie progressive » dans leur législation. L’approche de chaque pays, qu’il s’agisse d’abaisser l’âge du consentement dans des circonstances spécifiques (Mexique), d’établir des tranches d’âge différenciées avec divers degrés d’autonomie (Argentine) ou de lever complètement les restrictions fondées sur l’âge (Colombie), offre un mécanisme différent pour accroître l’autonomie des jeunes dans la prise de décision relative à l’avortement. L’article examine également comment un pays qui a récemment connu un recul en matière de droits à l’avortement, c’est-à-dire les États-Unis, a aussi restreint la capacité des jeunes à consentir de manière autonome à l’avortement et a renforcé l’implication des parents dans ces décisions. Avec une analyse comparée des cadres juridiques de ces quatre pays, l’article conclut que des changements radicaux dans la législation et les politiques relatives à l’avortement, qu’ils soient de nature progressiste ou régressive, peuvent altérer profondément la capacité des jeunes à consentir à un avortement et à y accéder. Il propose aussi une série de recommandations pour encadrer la prise de décision des jeunes en matière d’avortement, notamment en fondant les lois et politiques pertinentes sur les principes internationaux relatifs aux droits humains et les reliant aux lois et politiques visant à protéger les droits des jeunes en général.

## Resumen

Este artículo analiza los marcos legislativos nacionales que regulan el acceso de las personas jóvenes al aborto, y se centra en la manera en que los diferentes enfoques de facultad decisoria pueden facilitar u obstruir el acceso a los servicios. Se examina la manera en que los países que recientemente liberalizaron sus leyes sobre aborto, es decir, Colombia, Argentina y México, han reconocido la necesidad de reducir la edad de consentimiento a menos de 18 y de integrar en su legislación los principios de “las capacidades evolutivas del niño” o “autonomía progresiva”. El enfoque de cada país ya sea reducir la edad de consentimiento en circunstancias específicas (México), establecer grupos de edad diferenciados con diversos grados de autonomía (Argentina) o eliminar por completo las restricciones basadas en la edad (Colombia), ofrece un mecanismo diferente para aumentar la autonomía de las personas jóvenes en la toma de decisiones sobre aborto. Además, el artículo considera la manera en que un país que ha experimentado una regresión reciente del derecho al aborto, es decir, Estados Unidos, también ha revertido la capacidad de la juventud para consentir autónomamente en recibir servicios de aborto y ha aumentado la participación de los padres en tales decisiones. Mediante un análisis comparativo de los marcos legislativos de estos cuatro países, el artículo concluye que los cambios radicales a las leyes y políticas sobre aborto ya sean de naturaleza progresista o regresiva, pueden alterar fundamentalmente la capacidad de la juventud para acceder a tales servicios y dar su consentimiento para recibirlos. Se ofrece una serie de recomendaciones para regular la toma de decisiones sobre aborto por la juventud, tales como basar las leyes y políticas pertinentes en los principios internacionales de derechos humanos y vincularlas con leyes y políticas orientadas a proteger los derechos de las personas jóvenes en general.

## Introduction

Over the past three decades, legislatures and high courts in many countries have reformed their abortion laws towards decriminalising abortion.^1^ Between 1994 and 2023, at least 60 countries and territories liberalised their abortion laws.^1^ In contrast, only a handful of countries have decreased the legal grounds on which pregnant people can access abortion services, namely El Salvador (1998), Nicaragua (2006), Poland (2020), and the United States (2022).^1^ These broader trends have affected many aspects of abortion law and policy, including young people's ability to make decisions about abortion care.

Historically, legal systems around the world have presumed that young people lack the capacity to make decisions without adult involvement. In the medical decision-making context, this presumed decisional incapacity means that consent is required from a parent or other legal guardian for most medical evaluation, procedures, treatment, and research involving young people below a certain age.*^,^^2,3^ Other actors, including the state, health providers, and educators, can play a role in young people's decision-making, particularly when a young person and their parent(s) or other legal guardians disagree.

Medical and legal scholars,^†^^,^^4–6^ as well as international organisations (e.g. World Health Organization, Committee on the Rights of the Child) and experts (e.g. Special Rapporteur on the right to health), however, have long recognised that patient autonomy is a core tenet of medical decision-making, including when patients are young people. They have underscored, moreover, the limitations and challenges associated with establishing 18 years of age as a strict cut-off for independent medical decision-making. International human rights law, for example, has sought to address the discrepancy between national legal systems’ historical treatment of young people's decision-making capacity and more recent understandings of young people's mental and emotional development through the establishment of new principles, such as the evolving capacities of the child.^7–9^ The World Health Organization (WHO) has consistently grounded its approach to young people's medical decision-making in human rights, maintaining that young people have a right to participate in and express their views on decisions regarding their health, including their sexual and reproductive health.^10,11^

The presumption of young people's incapacity extends to decision-making around sexual and reproductive health services, particularly abortion. Some countries that recently liberalised their abortion laws also increased young people's autonomy in abortion decision-making in the process. Between 2020 and 2022, as part of their legal reform processes, Colombia, Argentina, and Mexico adopted new approaches to regulating young people's access to abortion that better acknowledge their decision-making capacity. The United States, conversely, has seen an uptick in efforts to increase parental involvement and decrease patient autonomy in the context of abortion decision-making involving young people in the wake of the U.S. Supreme Court's 2022 decision to eliminate the federal right to abortion. This paper analyses the recent experiences of these four countries in depth to uplift how broader trends related to abortion law and policy affect young people's abortion decision-making capacity in particular.

## Methodology

This article features a comparative law and policy analysis of the decision-making capacity of young people related to abortion across four national legal frameworks, namely Colombia, Argentina, Mexico, and the United States. It analyses the approach taken in these countries, which were chosen specifically because of their geographic proximity, variable legal approaches, number of relevant primary legal sources, and recent changes to abortion laws.

For each country, we identified the jurisdiction's principal primary legal sources for in-depth analysis (see Supplementary Table S1 for the full list of primary legal sources analysed). We organised the primary legal sources into the following categories: constitutions, case law (judicial decisions), statutes (codes, laws, and resolutions), and regulations (policies, protocols, guidelines, and agreements). Primary legal sources were designated as “principal” if they established an original legal standard related to young people's decision-making or applied such a standard to either medical decision-making in general or abortion decision-making in particular. The number of principal primary legal sources selected per country ranged from six (Argentina) to 11 (Mexico). Four of the authors, who are bilingual in English and Spanish, carried out the analysis of the principal primary legal sources and translated the direct citations included in the article from Spanish to English as part of the drafting process.

The legal analysis also briefly considers the establishment and evolution of relevant international human rights law principles, including evolving capacities and progressive autonomy. The international human rights analysis is based on a comprehensive scoping of primary legal sources, focusing on treaty monitoring and regional bodies’ statements and recommendations on the gradual attainment of young people's decision-making capacity. Treaty monitoring bodies included the Committees on the rights of the child, on economic, social and cultural rights, and on the elimination of all forms of discrimination against women, and statements and recommendations included in general comments/recommendations and concluding observations. Regional bodies included the Inter-American Court of Human Rights (“IACtHR” or “the Court”). We then identified and analysed priority standards put forward by treaty monitoring bodies, focusing on the standards most relevant for the abortion decision-making context. See Supplementary Table S2 for the full list of primary legal sources analysed.

In each jurisdiction, our analysis of principal primary legal sources relied on a close reading of the text of each source, as well as consultation of relevant secondary sources (e.g. academic articles, reports by local civil society organisations). Across each of the countries considered as part of the comparative analysis, we considered the following criteria: age of consent, factors for capacity to consent, level of parental involvement, and reliance on international human rights standards. We analysed the legal impact of primary legal sources based on their recency, normative force, and their relevance to adolescents’ access to abortion care.

The legal research carried out to inform this article derives from a larger research project, Beyond Borders, which seeks to bring abortion law and policy experiences from around the world to the United States. That project featured a one-way desk review of the principal primary legal sources that govern young people's abortion decision-making in a greater number of countries and regions. The report “Regulation of Young People's Abortion Decision-Making”^12^ forms part of a series, which aims to increase visibility of approaches used to expand access to abortion care around the world among decision-makers and advocates in the United States and situate the United States within the broader comparative context.

### Reflexivity

The article has five authors, four of whom are bilingual in English and Spanish, representing a mix of geographies and career stages. All bring personal or professional perspectives rooted in the Global South or Latin America, and have longstanding experience in the fields of reproductive rights and human rights. All authors identify as cisgender women and are currently living and working in the United States, Latin America, or Europe. None of the authors are involved in decision-making or advocacy in the United States.

## Results

### Key principles in international human rights law

International human rights law has referenced a series of rights and principles in developing normative standards and recommendations regarding young people's abortion decision-making capacity. Since the 1980s, various human rights bodies have recognised the gradual attainment of decision-making capacity among young people and, in some cases, have called on states to reform laws requiring parental involvement in abortion decision-making.^12^ These standards have relied primarily on the “evolving capacities of the child” and “progressive autonomy” principles and the rights to health and privacy.

### Evolving capacities of the child

The 1989 United Nations Convention on the Rights of the Child (“CRC”) was the first international human rights treaty to acknowledge children as distinct rights holders. Article 5 of the CRC establishes that children have “evolving capacities,” meaning that their capacities evolve as they grow and develop. The evolving capacities principle establishes that, as a result, parents should adapt their direction and guidance accordingly to enable their children to take on a greater role and responsibility in exercising their own rights as they move through childhood and adolescence into adulthood.^13^ This principle is also closely tied to the CRC's general principles of non-discrimination, best interests of the child, right to development, and the right to be heard.

The Committee on the Rights of the Child (“CRC Committee”), the UN treaty body charged with interpreting and enforcing the obligations set out in the CRC, elaborated on Article 5 in successive General Comments. It has stated that “[t]he evolving capacities should be seen as a positive and enabling process, not an excuse for authoritarian practices that restrict children's autonomy and self-expression, and which are often inaccurately justified by pointing to children's relative immaturity.”^7^ It also stressed that soliciting children's views and giving them increasing weight is central to assessing their best interests, and neither parental authority nor cultural or religious traditions should be invoked to legitimise harmful practices or discriminatory restrictions, particularly against girls.^7^

Accordingly, the CRC Committee has provided that States should review and consider allowing children to consent to certain medical treatments and interventions without the permission of a parent, caregiver, or guardian, including sexual and reproductive health services (e.g. HIV testing and safe abortion) and mental health care.^8,14^ As recently as 2016, the CRC Committee recommended a presumption that adolescents are competent to access sexual and reproductive health services and stressed that barriers such as third-party consent are impermissible. It further urged States to guarantee pregnant adolescents’ best interests by ensuring their views are heard in abortion-related decisions, a position reiterated in concluding observations to Russia (2024),^15^ Djibouti (2022),^16^ Palestine (2020),^17^ and Ireland (2016).^18^

Other UN treaty bodies have reinforced the CRC Committee's recognition that young people gradually attain decision-making capacity. The Committee on the Elimination of Discrimination against Women, in its General Recommendation No. 24 on women and health, affirms that States should ensure adolescents’ access to sexual and reproductive health without barriers such as parental authorisation.^19^

#### Progressive autonomy

The Inter-American human rights system has developed its own principle to account for young people's gradual attainment of decision-making capacity, namely “progressive autonomy.” While the Inter-American Court of Human Rights (“the Court” or “the IACtHR”) has recognised since 2002 that children are rights-holders,^20^ it was not until 2011 that the Court explicitly addressed the progressive exercise of their rights. In 2011, the Court held that children must be able to exercise the right to self-determination in a progressive manner.^‡^^21^ According to the Court:

*While children are subject to human rights, this right implies the possibility of all human beings to self-determination and to freely choose the circumstances and options regarding their existence. In the case of children, they exercise this right in a progressive manner in the sense that the minor of age develops a greater level of personal autonomy with time […].^21^*

One year later, the IACtHR (“the Court” hereafter) held that “children exercise their rights progressively as they develop a greater level of personal autonomy.”^§^^22^ The Court further emphasised that authorities must “take into account the specific conditions of the child and his or her best interests to decide on the child's participation, as appropriate, in determining his or her rights.”^22^ Drawing on General Comment No. 12 of the CRC Committee, the Court underscored that the right of each child to express his or her views in all matters affecting them also includes the subsequent right to have those views taken into consideration according to age and maturity.^22^ Similarly, in 2013, the Court held that the principles of the best interests of the child and progressive autonomy require both the maximum satisfaction of children's rights and the minimum restriction on their exercise.**^23^ Building on these precedents, as recently as 2021, the Court cited the CRC Committee in reaffirming that “children exercise their rights progressively as they develop a greater level of personal autonomy.”^††^^24^

In 2020, the Court recognised that young people may exercise other rights contained in the American Convention on Human Rights in a progressive manner.^‡‡^^25^ It held that the rights to personal integrity (Article 5) and private life (Article 11) protect “sexual freedom and control over one's own body” and “may be exercised by adolescents in the measure that they develop the capacity and maturity to do so.”^25^ The Court also noted in a footnote that, according to the CRC Committee, levels of comprehension in children “are not uniformly linked to their biological age” and “that information, experience, social environment, cultural expectations and the level of support received contribute [to] the development of the child's capacity to form an opinion.”^25^ Accordingly, factors influencing the decision-making of children and adolescents must be considered “when trying to ensure a proper balance between respect for the progressive autonomy and appropriate levels of protection.”^25^

Both international and domestic bodies play a role in operationalising international human rights treaties. Generally speaking, UN treaty bodies enforce and oversee States Parties’ compliance with treaty obligations through periodic reporting procedures and, where recognised, individual communications mechanisms.^§§^ Enforcement mechanisms and authority vary by regional human rights system. In the Inter-American Human Rights System, the Inter-American Commission on Human Rights monitors compliance through country and thematic reports, merits reports containing recommendations, and the issuance of precautionary measures. The Court, in turn, issues binding judgments and provisional measures for States Parties that have accepted its jurisdiction.

While international human rights treaties are legally binding upon ratification by States Parties, the relative domestic authority of these instruments varies across legal systems. All Latin America countries ratified the CRC in the early 1990s, including Argentina (1994), Colombia (1991), and Mexico (1990).^26^ Of those countries, only Argentina has ratified the CRC's 2015 optional protocol, which enables individuals to submit individual complaints against their respective government to the CRC Committee for alleged rights violations. Similarly, Argentina, Colombia, and Mexico have ratified the American Convention on Human Rights and recognised the IACtHR's authority to hear disputes brought by individuals from their respective countries.^27^ In all three countries, international human rights treaties form part of the national constitutional framework and are directly enforceable by domestic courts. In fact, human rights norms have the same hierarchical status as the constitution, subject to varying caveats.*** Authoritative interpretations and recommendations issued by treaty bodies inform domestic understanding of treaty obligations in all three countries, while the relative domestic authority of the IACtHR's decisions varies by country.^†††^ In Mexico, IACtHR's interpretative criteria are binding on domestic courts, regardless of whether Mexico was a party to the specific case.

Conversely, the CRC's obligations are not legally binding upon the United States, as it is the only remaining country that has yet to ratify the treaty. And while the United States has signed the American Convention on Human Rights, it has yet to ratify it and has not accepted the jurisdiction of the IACtHR.

Accordingly, the degree to which national legal frameworks reference or explicitly integrate either the evolving capacities or progressive autonomy principles, including in the context of abortion law and policy, varies by country.

### Case studies

#### Colombia: no age of consent

In Colombia, the decriminalisation of abortion has unfolded throughout 25 years of Constitutional Court jurisprudence,^28^ culminating in the landmark 2022 ruling C-055-2022 that decriminalised abortion through the first 24 weeks of pregnancy. In parallel, the Court has developed the standards governing children's and adolescents’ ability to consent to and access health services, including abortion.

Colombia's Constitution contains a series of fundamental rights that serve as the basis for the constitutional jurisprudence recognising young people's abortion decision-making autonomy. Article 44 of the Constitution establishes that “life, physical integrity, health and social security, a balanced diet, their name and citizenship, to have a family and not be separated from it, care and love, instruction and culture, recreation, and the free expression of their opinions” are basic rights of children.^29^ It further establishes that the family and the State have an obligation to assist and protect children “in order to guarantee their harmonious and integral development and the full exercise of their rights.” It additionally states that the rights of children “take precedence over the rights of others.”^29^ With respect to adolescents, Article 45 of the Constitution provides that “adolescents are entitled to protection and integral development,” and further establishes that both the State and society must “ensure the active participation of adolescents in public and private institutions responsible for their protection, education, and progress.”^29^

The Children and Adolescents Code further defines parental obligations and responsibilities in the upbringing of boys, girls, and adolescents. Specifically, Article 14 considers parental responsibility as the:

*[O]bligation inherent to the guidance, care, accompaniment and upbringing of boys, girls and adolescents. This includes the shared and joint responsibility of the father and mother to ensure that children and adolescents can achieve the maximum level of satisfaction of their rights.^30^*

In relation to sexual and reproductive health, the Code explicitly establishes that access to these services must be guaranteed as a special obligation of the social security health system.

In a series of cases decided in the 1990s and early 2000s,^‡‡‡^ the Colombian Constitutional Court considered how these rights are implicated in the context of young people's medical decision-making and established a normative basis for eventually eliminating age-based cut-offs for abortion decision-making. Drawing on the constitutional rights to dignity, autonomy, and the free development of personality, the Court held that chronological age, by itself, is not a valid determinant of legal capacity or decision-making autonomy. Rather, age may operate only as a reference point for evaluating a minor's intellectual and emotional maturity. Accordingly, the Court emphasised that minors of the same age may “show different capacities for self-determination, and therefore may enjoy different protections of the right to the free development of the individual.”^31^ The Court considered that the legal protection afforded to the right to free development of personality increases “as the ability of self-determination of the minor increases”^31,32^ and, as a result, “the higher the degree of intellectual capacity, the lesser the legitimacy of interventions into the decisions of the minor.”^31,32^

When decriminalising abortion under three circumstances in 2006, the Court also considered the constitutionality of Article 123 of the Criminal Code, which prohibited any abortion “performed on a woman of less than 14 years of age.” The Court ruled that this presumption was unconstitutional because it nullified the legal effect of a minor's consent and, in doing so, disregarded the human dignity, autonomy, and free development of personality of pregnant minors under 14.^33^ The Court held that while the Colombian legislature could “establish rules in the future regarding representation of minors or the assertion of minors’ rights,” those rules cannot invalidate the consent of a minor under 14 years of age.^33^

In 2009, the Court explicitly named parental consent an impermissible requirement for obtaining an abortion, characterising “preventing girls under 14 from providing informed consent when their parents disagree” as an unconstitutional barrier to legal abortion.^34^ The Court reiterated this approach in 2016, stressing that only the minor's “consent is required to undergo a voluntary termination of pregnancy.”^35^ Similarly, in 2018, the Court stated:

*Minors are full holders of the right to free development of personality and to that extent enjoy full capacity to consent to treatments and interventions on their bodies that affect their sexual and reproductive development, including voluntary termination of pregnancy. Obstacles or additional barriers should not be imposed when their parents or legal representatives do not agree with the consent given for this purpose.^36^*

Taken together, these decisions demonstrate the Court's gradual consolidation of a doctrine grounded in the evolving capacities and progressive autonomy principles. The judicial precedents established the normative foundation for not only dismantling both age-based and parental involvement-related barriers, but also requiring that determinations about decision-making be tied to an individual's capacity for self-determination.

This jurisprudence has also informed the development of Colombia's administrative regulations on access to abortion for adolescents. Colombia adopted and then updated a resolution regulating the provision of abortion in 2018 and 2023, respectively.^§§§^ The resolution (No. 051/2023) includes provisions that integrate the standards adopted by the Constitutional Court related to the ability of all young people, regardless of age, to consent to and access abortion without parental involvement.^37^ It states that:

*Girls under 14 years old can exercise their right to VTP [voluntary termination of pregnancy] autonomously. Their wish to terminate or continue with the pregnancy takes precedence over the wishes of their parents or legal representatives, even if they do not agree with their decision.^37^*

The resolution references the concept of “substitute consent” but stresses that in cases involving abortion, it is not decisive and that only the minor's decision is valid.^37^

In February 2025, the Ministry of Health passed a resolution on measures aimed at guaranteeing “access, autonomy, and informed consent for girls, boys, and adolescents in healthcare.”^38^ The resolution seeks to integrate the principles of evolving capacities and progressive autonomy established by both the CRC Committee and Colombia's Constitutional Court into healthcare settings. Notably, the resolution introduces six rules to guide the “intensity of support” provided by adults to children and adolescents related to medical decision-making, addressing factors such as the level of risk of the procedure or treatment, disagreement between minors and legal representatives, and situations where it is not possible to determine the minor's decision.^38^

#### Argentina: age brackets

In Argentina, legislators adopted an “age-range” or “age brackets” framework, in which different age ranges are associated with different levels of decision-making capacity, recognising the gradual attainment of decision-making capacity among young people. While this approach emerged initially in provisions governing general medical decision-making capacity, it has been integrated into the county's new abortion law.

Argentina first integrated the evolving capacities and progressive autonomy principles into its national legal framework with the adoption of Law No. 26.061, the “Comprehensive Protection of the Rights of Children and Adolescents,” in 2005.^39^ It establishes that children and adolescents must be heard in all judicial or administrative proceedings affecting them. It guarantees their right to express their views before the competent authority, to have those views be given primary consideration, to receive specialised legal assistance, and to appeal adverse decisions.^39^ In 2009, the Patient Rights Act integrated the principle of progressive autonomy into its provision on freedom of choice, stating that children and adolescents have the right to “intervene for the purposes of making decisions about medical or biological therapies or procedures that involve their life or health.”^40^

Most notably, in 2015, Argentina's legislature reformed its Civil and Commercial Code with the intent to align the country with its obligations under international human rights law, including the CRC's approach to young people's decision-making capacity. In doing so, Article 26 of the Code incorporated an age brackets framework to govern young people's consent for different types of medical treatment based on level of risk. The provision states that:

*Regarding consent to medical treatment, it is presumed that adolescents between 13–16 have the capacity to decide for themselves regarding non-invasive treatments that do not compromise health, or carry a serious risk to life or physical integrity. If treatments are invasive or do compromise health or life, the adolescent aged 13–16 must give consent with the assistance of their parents. Conflicts are resolved based on the medical opinion of the provider, based on the minor's best interest. From age 16, the adolescent is considered an adult for decisions related to the care of their own body.^41^*

Article 639, which addresses parental responsibility, also establishes that parents must respect the child's best interests, progressive autonomy, and right to be heard, all proportionate to age and maturity.^41^ These three laws formally integrated gradual attainment of medical decision-making capacity for young people into Argentina's national legal framework.

Argentina also integrated the age-bracket framework into its 2019^42^ and 2022^43^ abortion protocols,**** which directly applied Article 26 of the Civil and Commercial Code to abortion services.^41^ Under these protocols, adolescents aged 16 and above are treated as adults for the purposes of consenting to abortion, requiring no parental involvement. Those aged 13–16 may consent independently as long as the abortion does not pose a serious risk to their life or health. Where such risk exists, parental participation is required. For children under 13, abortion decisions are to be made through a shared decision-making process involving parents or guardians, consistent with the principle of progressive autonomy.

Both protocols also provide mechanisms for resolving conflicts where parental involvement is required but withheld. If parents or guardians refuse to support the child's decision, the adolescent may proceed with the support of another family member. Where this is not possible, the healthcare team is responsible for resolving the conflict by prioritising the child's best interests, the principle of non-substitution of consent, and the child's demonstrated decision-making capacity.^43^

#### Mexico: lower age of consent

The legal age of consent for most abortions in Mexico is formally set at 18. However, based on the recognition of children's rights and the principle of evolving capacities in technical standards and Supreme Court rulings, girls and adolescents have been recognised as having the right to access sexual and reproductive health services without parental involvement and to obtain abortion care in cases of sexual violence from the age of 12.

Children's rights in Mexico have constitutional standing. Article 4 of the federal constitution establishes the state's obligation to uphold the best interests of the child and to guarantee their rights.^44^ Moreover, under Mexico's conventionality control framework, various international treaties containing provisions on children's rights are applicable, including the Convention on the Rights of the Child and the American Convention on Human Rights.^45^ This principle is further developed in the General Law on the Rights of Children and Adolescents, which provides that “the best interests of the child must be considered a primary factor in decision-making” in all matters affecting children and adolescents.^46^ The law also enshrines the right to health, specifically including preventive care and sexual and reproductive health services.^46^ The General Health Law reinforces this protection, requiring health care providers to implement support and reasonable accommodations when treating children and adolescents, to ensure that their will and preferences are taken into account.^47^

Building on these precedents, in 2015, the Ministry of Health issued the Official Mexican Standard^††††^ on health care of persons between 10 and 19 years of age.^48^ The rationale for this rule emphasised that, in matters concerning the human rights of children and adolescents, a specialised constitutional and international framework must guide the design, implementation, monitoring, and evaluation of public policies affecting children.^48^ The rule requires counselling and guidance on family planning and sexual and reproductive health, tailored to children's evolving cognitive development and maturity.^48^ Care may be requested directly by girls and adolescents, who may choose whether or not to be accompanied by a parent or guardian. While the rule recognises pregnancies among minors as high risk and requires providers to identify risk factors and screen for signs of sexual violence or abuse, it does not specify an age of consent for abortion care.^48^

In Mexico, both federal and state law play a role in the regulation of abortion care. While Supreme Court jurisprudence established that the criminalisation of abortion in early pregnancy violated the country's federal constitution, state legislatures nonetheless needed to take action to decriminalise abortion in their respective criminal codes. As of early 2026, only 25 of the country's 32 states had decriminalised abortion during the first trimester in accordance with Supreme Court precedents.^‡‡‡‡^^49^

At the same time, federal health legislation establishes the overarching framework governing the organisation and delivery of public health services nationwide. Binding Official Mexican Standards (NOMs) and institutional guidelines issued by federal health entities, including the Institute for Social Security and Services for State Workers (ISSSTE), regulate particular aspects of abortion care within that system.

In 2016, the Official Mexican Standard on medical care in cases of family and sexual violence (NOM-046) was amended to lower the age of consent for abortion, establishing that girls aged 12 and older who are survivors of sexual violence may access abortion without parental or guardian authorisation.^50^ For those under 12, parental or guardian authorisation is required.^50^

This reform was challenged by members of the Aguascalientes^51^ and Baja California^52^ legislatures, who argued that it intruded on state jurisdiction to legislate parental authority and its effects on minors, citing provisions of the state civil code on parental authority.^51^ The Supreme Court upheld NOM-046, relying on the constitutional framework and international human rights standards. It held that:

*[R]equiring judicial authorisation for health services to provide abortion care, or requiring parental authorisation, in fact constitutes a form of violence and discrimination against girls and women, or persons with the capacity to become pregnant, who are victims of rape.^52^*

The Court reasoned that demanding authorisation to access services uniquely required by these populations, such as abortion care, and imposing barriers that restrict or limit access, amount to acts of discrimination and violations of the right to equality.^52^

The Court explicitly referred to the CRC's principle of evolving capacities and its protection of the right to health.^52^ It underscored the link between evolving capacities and the right to health, stressing that children's ability to assume responsibility and make decisions affecting their lives, including medical treatments and interventions, gradually increases.^51^ Citing its previous jurisprudence, the Court described childhood as a cumulative developmental process:

*The stages of a child's development are cumulative; each one impacts subsequent stages, influencing the child's health, potential, risks, and opportunities. Understanding their life trajectory is crucial for safeguarding their right to the highest possible standard of physical and mental health*.^51^

Accordingly, children's right to health, including sexual and reproductive health, encompasses “the right to control one's own health and body” as capacities and maturity evolve.^51^

The Court also reflected on Mexico's conception of parental authority, which balances the need to protect children with the recognition of their progressive autonomy.^51^ While “[c]omprehensive protection of minors is a constitutional mandate imposed on both parents and public authorities,” children must also “be recognized as persons and rights holders with progressively increasing capacity to exercise those rights as they mature.”^51^ According to the Court, allowing survivors of sexual violence between the ages of 12 and 18 to access abortion without parental consent is essential to safeguarding the best interests of the child and consistent with the authorities’ duty to protect survivors and to “take special measures aimed at reducing the direct and indirect effects of traumatic experiences and ensuring the healthy and harmonious development of their personality in the future.” Crucially, the Court emphasised that Article 4 of the Mexican constitution, which enshrines the best interests of the child principle, serves as a limit on parental rights:

*[J]udicial authorities must move beyond the old conception of parental authority as absolute parental power. Today, parental authority is not a right of the parents, but a function entrusted to them for the benefit of the children, directed at their protection, education, and comprehensive development, with the child's best interests always prevailing in the parent–child relationship. Public authorities are increasingly vigilant in ensuring this principle.^51^*

The Supreme Court has also addressed other barriers to abortion access for minors who were victims of sexual violence, including the 90-day gestational limit and reporting requirements. In 2021, the Court ruled that the 90-day gestational limit was unconstitutional and violated children's rights, given that many young people may not even know or realise they are pregnant in the early stages.^53^ In another case in 2022, involving a child survivor of rape, the Court held that requiring survivors to file a criminal complaint before becoming aware of the pregnancy was contrary to the best interests of the child, as it created secondary victimisation and infringed upon other human rights.^54^

In 2022, the Mexican Ministry of Health issued Technical Guidelines on Safe Abortion Care, which affirm that “children and adolescents enjoy progressive autonomy grounded in individual freedom and the capacity of each person to regulate their rights and obligations.”^55^ The guidelines incorporated the standards upheld in NOM-046 and by the Supreme Court, reaffirming that girls and adolescents aged 12 and older may request and consent to abortion in cases of pregnancies resulting from sexual violence without parental involvement.^55^ For those under 12, abortion care must be requested “through” a parent or guardian; if none is available, a representative of the child protection authority may make the request in accordance with the child's best interests. Abortion services, moreover, must take into account the best interests of the child, the principle of good faith, and progressive autonomy, as well as recognition of the evolving capacities of children and adolescents.^55^ These standards are binding on all health care providers and have gradually been incorporated into clinical directives governing abortion care more broadly, not only in cases of sexual violence.

In January 2025, the ISSSTE, a federal agency which provides social security and health care services to federal government employees, issued a general act guaranteeing procedures for abortion in the agency's medical units. Building on already existing provisions, the act provided more concrete guidance regarding the requirement that young people under 12 must be accompanied by a representative during the provision of abortion services.^56^ Notably, the representative must be chosen and expressly endorsed by the adolescent, and the absence of accompaniment cannot be invoked as a justification for denying medical care. Moreover, where there is a conflict between the decision of the girl or adolescent and her legal representative, health authorities are required to provide the former with truthful, objective, and impartial information regarding the risks and consequences of undergoing or not undergoing an abortion, and “adhere to the decision to which she inclines” in order to reach a determination.^56^

#### United States: increased parental involvement

In the United States, whether a young person can consent to medical care, including abortion care, is governed largely by state rather than federal law, and there is considerable variation among states’ relevant laws. While states’ laws generally presume that young people are incapable of consenting to medical treatment until the age of majority (i.e. 18), minor consent laws permit those under the age of majority to consent to specific services (e.g. pregnancy-related care, contraception, abortion) or based on status (e.g. pregnancy, marriage, emancipation). While some states have eliminated parental involvement requirements from abortion laws, the vast majority (37 states) still require some type of parental involvement in a young person's decision to have an abortion, ranging from written consent to notification.^57,58^ Parental involvement requirements in the United States most often apply to young people under 18, but some statutes apply only to those under 17 (e.g. South Carolina) or 16 (e.g. Massachusetts, Montana).^58^

Despite state variation, U.S. Supreme Court jurisprudence has greatly affected the extent to which states can restrict young people's ability to consent to abortion care, with recent jurisprudence overturning prior protections and opening the door to further restrictions. The Supreme Court's landmark *Roe v. Wade* decision established a federal right to abortion under the constitution's right to privacy but failed to address to what extent that right applies to young people. When states then began requiring parental involvement in young people's abortion decision-making, the Supreme Court issued a series of decisions regarding the constitutionality of such requirements.

As early as 1976, the Supreme Court held that minors, like adults, hold constitutionally-protected rights, and that states cannot delegate to parents a veto power that states themselves do not have.^58,59^ However, in 1979, the Court also recognised the “guiding role of parents” in light of minors’ “inability to make critical decisions in an informed and mature manner.”^60^ Accordingly, the Court determined that states have a special interest in encouraging unmarried, pregnant minors to seek their parents’ guidance in their abortion decision. Seeking to balance this state interest against the unconstitutional delegation of veto power to parents, the Court ruled that parental involvement laws must include a confidential, expeditious, and non-adversarial judicial bypass option.^60^ The Supreme Court's general framework for the judicial bypass mechanism also emerged from this decision, requiring judges to grant minors’ petitions if they determine that the minor is well-informed and mature or that an abortion would be in the minor's best interests.^60,61^

In the years that followed, the vast majority of states with parental involvement requirements integrated a judicial bypass mechanism into their respective laws. While the specific process, timing, and criteria for obtaining judicial bypass have varied by state, critics of the judicial bypass mechanism have stressed that it can threaten young people's privacy and delay access to abortion care.^61^ It also affords wide discretion to judges to assess young people's maturity and best interests, leading to highly subjective rulings sometimes based on a judge's personal beliefs rather than objective factors.^62^ Despite its limitations, the judicial bypass mechanism can mitigate the harms caused by parental involvement laws and facilitate access to abortion care for some young people.^61^

Other types of exceptions to parental involvement laws also exist – either alongside or in place of the judicial bypass mechanism. Nearly all parental involvement requirements include an exception for medical emergencies.^63^ Some states allow the consent of a non-parent family member (e.g. Colorado, North Carolina), doctor or mental health professional (e.g. Delaware, Oregon), or other adult (e.g. Virginia) as a substitute for parental consent.^57,61,63^ Maryland has adopted the mature minor doctrine.^§§§§^^63^

In the wake of the Supreme Court's decision to overturn the federal right to abortion in *Dobbs v. Jackson Women's Health Organization*, young people's abortion decision-making has come under attack. In *Dobbs*, the Court afforded states the authority to ban, restrict, or protect abortion access, and various cases brought before courts since 2022 raise the question of whether the constitution still requires parental involvement laws to contain a judicial bypass mechanism. In a Florida case,***** an appellate court ruled in 2025 that the state's judicial bypass mechanism constituted an unconstitutional violation of parental rights.^64^

At least two states, namely Missouri and Montana, filed petitions for writs of *certiorari* with the Supreme Court, asking it to weigh in on the constitutionality of judicial bypass post-*Dobbs*.^65^ While Missouri's petition asked whether a right to judicial bypass still exists, Montana's petition went further, asking whether judicial-bypass provisions are constitutional. According to Montana, absent a minor's countervailing fundamental right to choose abortion, parents’ constitutional right to direct the upbringing of their child supersedes other rights or interests. While the Court denied *cert* in both cases, some of the Justices arguably expressed an interest in reviewing similar cases in the future.^66^ Justice Alito indicated that the Montana case was merely a bad vehicle to answer the question on “[w]hether a parent's fundamental right to direct the care and custody of his or her children includes a right to know and participate in decisions concerning their minor child's medical care, including a minor's decision to seek an abortion.”^66^

In the wake of *Dobbs*, some states have taken other types of actions to reduce young people's abortion decision-making capacity. Some states without parental involvement laws are pushing to add parental notification requirements (e.g. Connecticut and Nevada), while others are attempting to increase the level of parental involvement required by moving from notification to consent (e.g. Delaware). Alternatively, various states (e.g. Idaho and Tennessee) have sought to criminalise “recruiting, harboring, or transporting” minors to help them access abortion in other states without parental consent.^67^ All efforts to curtail autonomous decision-making among young people related to abortion care and increase parental involvement in those decisions, in particular, are connected to a broader “parental rights” movement that has sought to increase parental involvement in their children's education, health decision-making, and expression of gender identity.^†††††^^68^

## Discussion

Countries that liberalised their abortion laws in recent years generally adopted laws and policies that increased young people's autonomy in abortion decision-making. Colombia did so by removing age-based restrictions altogether, Argentina did so by establishing differentiated age brackets with varying degrees of autonomy, and Mexico did so by lowering the age of consent in specific circumstances. While each country took a somewhat different approach to increasing decision-making capacity, they all referenced the limitations of strict age cut-offs, relied upon established statutory language or judicial precedent, and referenced international human rights law principles in the process.

Colombia's experience demonstrates how the evolving capacities and progressive autonomy principles can be relied upon to eliminate an age of consent for abortion, enabling young people to exercise fully autonomous decision-making related to abortion care. While Argentina does not go so far as to eliminate an age of consent, its age-bracket approach seeks to ground all young people's abortion decision-making in the principle of progressive autonomy, even in contexts where parents are involved in a shared decision-making process. Mexico's experience illustrates how incremental reforms, grounded in constitutional principles and international human rights law, have lowered the age of consent in specific circumstances. By recognising girls’ and adolescents’ evolving capacities, particularly in cases of sexual violence, the Supreme Court and health authorities have progressively limited parental authority and expanded young people's autonomy in abortion decision-making.

In contrast, the United States’ experience highlights the complexities of having varied approaches to young people's abortion decision-making among states. While there is relatively little research comparing these approaches or access to abortion for young people across them in practice,^‡‡‡‡‡^ experts have characterised the existence of a parental involvement requirement as the most consequential factor when it comes to young people's abortion access.^58^ Substituted consent by other adults, including judges, only goes so far in advancing young people's autonomy, particularly when compared with the removal or lowering of age cut-offs for abortion care. Moreover, following the elimination of the federal right to abortion in the United States, efforts to decrease young people's autonomy in abortion decision-making have gained momentum. In several cases, decision-makers have been persuaded by “parental rights” arguments and given little weight to young people's reproductive autonomy or other rights. The United States’ recent regression in abortion rights has clearly emboldened anti-abortion decision-makers and advocates to advance laws aimed at further decreasing young people's ability to autonomously consent to abortion care.

Regardless of each country's recent trajectory related to abortion law and policy, all four countries continue to acknowledge the need to carefully balance the principles of protection and autonomy in the context of young people's decision-making capacity. All three countries that increased autonomy in young people's abortion decision-making (Colombia, Argentina, and Mexico) account for possible cases of coerced abortion or insufficient maturity for independent decision-making by adopting safeguards, ranging from shared decision-making (Argentina) to non-directive counselling (Colombia) models.

The recent experiences of these four countries offer important insights for decision-makers and advocates advancing or responding to abortion law and policy reform efforts. First, international human rights law provides a powerful normative foundation for increasing young people's autonomy in abortion decision-making, clearly laying out various principles that can be relied on as part of national-level law and policy reform efforts. These principles may be particularly useful in national contexts with significant political or cultural resistance towards granting greater reproductive autonomy to young people.

Second, incremental change may be needed to pass laws and policies guaranteeing more autonomous abortion decision-making by young people at the national level. Colombia, Argentina, and Mexico, notably, all first integrated the evolving capacities or progressive autonomy principle in other national-level statutes and jurisprudence involving young people's decision-making related to health in general or other contexts altogether.

Finally, shifting the focus away from parental rights and towards children's rights is a necessary precondition for greater autonomy in abortion decision-making by young people. Other countries can learn not only from Colombia's, Argentina's, and Mexico's incorporation of international human rights principles and gradual approach to law and policy reform, but also from their legal system's establishment and development of specific fundamental rights for young people. These countervailing rights are crucial to ensuring that young people are truly centred in the decision-making processes that profoundly affect their lives, health, and wellbeing.

Accordingly, we offer three recommendations to decision-makers and advocates seeking opportunities to enhance young people's ability to autonomously consent to abortion care:

1. Eliminate or lower strict age cut-offs for young people seeking abortion care, or adopt age ranges for abortion decision-making capacity among young people reflecting their gradual attainment of the capacity to consent.
2. Ground national laws and policies governing young people's abortion or general medical decision-making in international human rights principles, particularly “evolving capacities of the child” or progressive autonomy.
3. Adopt or further strengthen laws and policies that lay out specific fundamental rights for young people, particularly their rights to development, participation, health, and privacy.

### Limitations

Given the variability of legal systems, frameworks, and traditions around the world, some of the insights and recommendations generated by this analysis may only be generalisable for the Americas region and would require further adaptation for application to other national contexts. For example, countries engage with international human rights law to varying degrees, and a lack of engagement may affect the feasibility of grounding national laws and policies in international human rights principles. The relative domestic authority of different primary legal sources, moreover, varies by legal system. The role of case law, in particular, in establishing and developing new legal standards depends on a specific country's legal tradition, as well as other factors. Finally, federalist legal systems (e.g. Mexico and the United States) differ significantly from non-federalist legal systems. The variation of relevant legal standards at the state level, as well as their interaction with federal law, may make it difficult to apply some insights generated by the Mexico and United States case studies to non-federal legal systems.

In addition, the existence of rights-based laws and policies does not guarantee access to abortion care in practice. Laws and policies must be effectively and equitably implemented in order to ensure young people are in fact able to make autonomous decisions about and access abortion care. Civil society organisations in Colombia, for example, have documented a range of legal and factual barriers to care in the years following the country's broad decriminalisation of abortion.^70^ Implementation, moreover, depends on a range of factors (e.g. political will, administrative capacity, dedicated funding, oversight mechanisms, etc.), many of which are affected or determined by rapidly changing political contexts. In Argentina, for example, provincial governments have assumed responsibility for the provision and financing of abortion care due to national budget cuts and administrative decentralisation in recent years, resulting in regional disparities in availability and quality of care. Abortion research from various national contexts, moreover, demonstrates that implementation-related challenges are more likely to disproportionately affect marginalised groups, including young people. However, an analysis of how laws and policies governing young people's abortion decision-making are implemented in practice in their respective national contexts is beyond the scope of this article.

## Conclusion

Sweeping changes to abortion law and policy – whether progressive or regressive in nature – can fundamentally alter young people's ability to consent to and access such care. The experiences of Colombia, Argentina, and Mexico underscore that enhancing young people's autonomy in abortion decision-making is consistent with emerging international human rights law standards. Each of these countries has incorporated the principle of evolving capacities or progressive autonomy, developed under international human rights law, into their national legal frameworks. Although the exact approach and legal mechanism vary by country, all three experiences reflect a deliberate effort to move away from rigid age cut-offs and towards individualised recognition of adolescents as rights holders. The experience of the United States, conversely, underscores the dangers associated with centring and prioritising parents’ rights and interests in laws and policies seeking to advance young people's sexual and reproductive health.

This comparative analysis of four countries’ legal frameworks underscores how different approaches to decision-making capacity can either facilitate or obstruct access to abortion care for young people. A rights-based approach to reproductive autonomy has the potential to be particularly transformative for young people, given the unique threats and barriers to essential sexual and reproductive health services they face based on their minority.

## Supplementary Material

Supplemental materials. Tables S1 and S2.

## Footnotes

* Informed consent requires patients to have: (1) the capacity to consent; (2) an understanding of all information relevant to the decision; and (3) the ability to exercise a voluntary decision free from coercion or manipulation.^2^

^†^
From a neuroscience perspective, scholars have noted that adolescents are capable of making adult-level, goal-oriented decisions, including those with long-term consequences, when placed in the appropriate context.^3^ Developmental science research has further established that by mid-adolescence, young people demonstrate decision-making capacities comparable to adults, particularly in areas such as working memory, logical reasoning, risk-benefit analysis, and the ability to anticipate the consequences of their actions.^4^ From a legal perspective, scholars have argued that creating a bright-line rule that rigidly separates childhood from adulthood, categorically denying children participation rights and other entitlements while at the same time allowing full accountability and punishment for their missteps, is problematic and inconsistent with international human rights standards.^6^

^‡^
*Gelman v. Uruguay* involved the forced disappearance of a pregnant student and the subsequent kidnapping of her child during Uruguay's military dictatorship.^21^

^§^
*Atala Riffo v. Chile* involved the discriminatory treatment and arbitrary interference with private and family life on the basis of the mother's sexual orientation.^22^

** *Mendoza v. Argentina* involved the imposition of life sentences on individuals for offenses committed while they were children, under a juvenile justice system that allowed them to be treated as adult offenders.^22^

^††^
*Furlan and Family v. Argentina* involved a child who suffered a serious injury while playing in an abandoned facility of the Argentinean Army.^23^

^‡‡^
*Paola Guzmán Albarracín v. Ecuador* involved a case of sexual violence committed against an adolescent girl.^25^

^§§^
Almost all treaty bodies review periodic reports submitted by States Parties on domestic implementation. Most may also consider individual complaints where States Parties have accepted the relevant procedure, and some are further authorized to conduct inquiries or examine inter-State communications.

*** In Argentina, in order to attain constitutional hierarchy, human rights treaties require a two-thirds vote of all the members of each House, after their approval by Congress. In Mexico, the human rights norms have the same hierarchical status as the constitution, unless the constitution expressly provides otherwise.

^†††^
In Colombia, the margin of appreciation for considering the interpretative criteria established by IACtHR decisions depends on whether Colombia is party to the specific case.

^‡‡‡^
The Colombian Constitutional Court has interpreted minors’ evolving capacities across several rulings, including Sentencia C-344/93, Sentencia T-477/95, Sentencia T-542/96, Sentencia T-474/96, and Sentencia SU-337/99.

^§§§^
This resolution references not only “progressive autonomy,” but also “contextual autonomy.”

**** The 2019 and 2022 protocols regulated the law decriminalising abortion under three circumstances and the law decriminalising abortion through 14 weeks of pregnancy, respectively.

^††††^
Mexican Official Standards (NOMs) are binding technical regulations that set the requirements processes or services must meet when they involve the safety and health of individuals, as well as provisions on terminology, compliance, and enforcement.

^‡‡‡‡^
The states that have decriminalized abortion are: Aguascalientes, Baja California, Baja California Sur, Campeche, Coahuila, Colima, Chiapas, Chihuahua, Mexico City, Guerrero, Hidalgo, Jalisco, Mexico State, Michoacán, Nayarit, Oaxaca, Puebla, Quintana Roo, San Luis Potosí, Sinaloa, Tabasco, Tlaxcala, Veracruz, Yucatán, and Zacatecas.

^§§§§^
A common law doctrine that permits young people below 18 who demonstrate sufficient maturity to provide informed consent for certain health services, including abortion.

***** *Doe v. Uthmeier* involved the judicial bypass proceeding for a 17-year-old seeking abortion without involving her parents. The young person had appealed the trial court's decision that she lacked the requisite maturity to access an abortion without parental involvement.

^†††††^
The anti-rights movement has relied on “parental rights” language to target not only inclusive education, but also gender-affirming care and contraception for minors.^68^

^‡‡‡‡‡^
One study indicates that lowering the age of consent reduced delays due to the elimination of the requirement to obtain parental consent or judicial bypass.^69^
