# Supplementary material for: Between protection and autonomy: a comparative analysis of legal approaches to abortion access for young people
Source: Sex Reprod Health Matters. 2026 Aug 7;33(1):2712800. doi: 10.1080/26410397.2026.2712800 (PMC13600281; doi:10.1080/26410397.2026.2712800)
Supplement: Supplementary file 1 [file ZRHM_A_2712800_SM9811.docx]

**Supplemental Materials**

Table S1: Primary Legal Sources Analyzed for Comparative Law Research

| **Country** | **Type** | **Title (Year)** |
| --- | --- | --- |
| Argentina | Statute | Law on Protection of Children’s & Adolescents’ Rights (2005) |
| Argentina | Statute | Patient Rights Act (2009) |
| Argentina | Statute | Civil and Commercial Code (2015) |
| Argentina | Regulation | Resolution on Sexual & Reproductive Health (2015) |
| Argentina | Regulation | Abortion Protocol (2019) |
| Argentina | Regulation | Abortion Protocol (2022) |
| Colombia | Constitution | Constitution of Colombia (1991) |
| Colombia | Case law | Decision SU-642 (1998) |
| Colombia | Case law | Decision SU-337 (1999) |
| Colombia | Case law | Decision C-355 (2006) |
| Colombia | Case law | Decision T-388 (2009) |
| Colombia | Case law | Decision T-697 (2016) |
| Colombia | Case law | Decision SU-096 (2018) |
| Colombia | Statute | Code of Children & Adolescents (2006) |
| Colombia | Regulation | Resolution 051 on Abortion (2018) |
| Colombia | Regulation | Resolution 051 on Abortion (2023) |
| Colombia | Regulation | Resolution 309 on Children’s & Adolescents’ Access, Autonomy and Consent in Health (2025) |
| Mexico | Constitution | Constitution of Mexico (1917, reformed 2026) |
| Mexico | Case law | Amparo 1049/2017 (2018) |
| Mexico | Case law | Amparo 8577/2019 (2020) |
| Mexico | Case law | Constitutional Controversy 45/2016 (2021) |
| Mexico | Case law | Constitutional Controversy 54/2016 (2021) |
| Mexico | Statute | General Law on Children’s & Adolescents’ Rights (2014) |
| Mexico | Statute | General Law on Family, Sexual, and Gender-Based Violence (2016) |
| Mexico | Statute | General Law on Health (2024) |
| Mexico | Regulation | Guidelines on Adolescents’ Health Care (2015) |
| Mexico | Regulation | Guidelines on Abortion (2022) |
| Mexico | Regulation | Agreement on Abortion Procedures (2025) |
| United States | Case law | *Planned Parenthood of Central Missouri v. Danforth (*1976) |
| United States | Case law | *Bellotti v. Baird* (1979) |
| United States | Case law | *Doe v. Uthmeier* (2025) |
| United States | Case law | *Chapman v. Doe* (2023) |
| United States | Case law | *Montana v. Planned Parenthood of Montana* (2025) |
| United States | Statute | Idaho Code Section 18-623 (2023) |
| United States | Statute | Tennessee Code Section 39-15-201 (2024) |
| United States | Statute | Parental consent/notification for abortion laws of all 50 states (2025) |

Table S2: Primary Legal Sources Analyzed for International Human Rights Law Research

| **Body** | **Type** | **Title (Year)** |
| --- | --- | --- |
|  | Treaty | American Convention on Human Rights (1969) |
|  | Treaty | United Nations Convention on the Rights of the Child (1989) |
| Committee on the Rights of the Child | General Comment | Statement on Article 5 of the Convention on the Rights of the Child (2003) |
| Committee on the Rights of the Child | General Comment | No. 7: Implementing Child Rights in Early Childhood (2005) |
| Committee on the Rights of the Child | General Comment | No. 12: The right of the child to be heard (2009) |
| Committee on the Rights of the Child | General Comment | No. 15: The Right of the Child to the Enjoyment of the Highest Attainable Standard of Health (2013) |
| Committee on the Rights of the Child | Concluding Observations | Combined Third and Fourth Periodic Reports of Ireland (2016) |
| Committee on the Rights of the Child | Concluding Observations | Initial Report of the State of Palestine (2020) |
| Committee on the Rights of the Child | Concluding Observations | Combined Third to Fifth Periodic Reports of Djibouti (2022) |
| Committee on the Rights of the Child | Concluding Observations | Combined Sixth and Seventh Periodic Reports of the Russian Federation (2024) |
| Committee on the Elimination of Discrimination against Women | General Recommendation | No. 24: Article 12 of the Convention (1999) |
| Inter-American Court of Human Rights | Case law | Advisory Opinion OC-17/2002 (2002) |
| Inter-American Court of Human Rights | Case law | *Gelman v. Uruguay* (2011) |
| Inter-American Court of Human Rights | Case law | *Atala Riffo & Daughters v. Chile* (2012) |
| Inter-American Court of Human Rights | Case law | *Mendoza & Others v. Argentina* (2013) |
| Inter-American Court of Human Rights | Case law | *Furlan and Family v. Argentina* (2021) |
| Inter-American Court of Human Rights | Case law | *Paola Guzmán Albarracín v. Ecuador* (2021) |
